# How long was it for you? Memories of the duration of the UK covid-19 lockdown

**DOI:** 10.1371/journal.pone.0271609

**Published:** 2022-07-15

**Authors:** Ruth S. Ogden, Andrea Piovesan

**Affiliations:** 1 School of Psychology, Liverpool John Moores University, Liverpool, United Kingdom; 2 Department of Psychology, Edge Hill University, Ormskirk, United Kingdom; University of Milan, ITALY

## Abstract

The covid-19 global pandemic has significantly impacted on the daily lives of people across the world. One consequence of this has been significant distortion to the speed at which time feels like it is passing during day-to-day life in comparison with prior to the pandemic. The current study sought to further understanding of the impact of the pandemic on temporal experience by exploring individual differences in the subjective length of the first 12 months of the pandemic in the UK. Using an online questionnaire, subjective judgments of the perceived length of the preceding 12 months were taken. In addition, measures of affect, task load and satisfaction with current levels of social interaction, physical activity, conformity with regulations, perceived covid risk and shielding status were taken. The results showed that only 9% of participants reported that the preceding 12 months felt like 12 months. The majority of participants (57%) reported that it felt like the pandemic had lasted for longer than 12 months, and this feeling was stronger for those who indicated greater levels of depression and anxiety, reduced physical activity, reduced satisfaction with social interaction and being advised to shield.

## Introduction

A well-documented consequence of the societal changes resulting from the covid-19 global pandemic is a change in the experience of the passage of time [1–9]. Studies conducted in the UK [1, 2], France [3, 4], Italy [5, 6], Argentina [7], Brazil [8], Iraq [9] and Uruguay [10] as well as a Europe wide study [10] have all demonstrated that during the pandemic, people experienced significant distortions to the speed at which time was passing in comparison with prior to the pandemic. In France [3, 4] and Italy [5, 6] citizens reported a slowing of the passage of time during the first weeks and months of the pandemic when social and physical distancing measures were enforced to reduce the spread of the virus. During a similar time, 80% of British people reported experiencing distortion to the passage of time, with 40% reporting that time was passing more quickly than normal and 40% reporting that it was passing more slowly than normal. A general slowing of the passage of time was also reported in Iraq 11 months into the pandemic during a period of minimal social and physical restrictions [9]. In Brazil, the pandemic was associated with an increased sense of time expansion and a reduction in time pressure [8]. In Argentina however, there was a tendency for people to experience an acceleration in time during the pandemic in comparison to before [7].

Despite some cross-cultural differences in the directionality of distortions to the passage of time during the pandemic, the factors which predicted temporal experience show some universality across countries and cultures. In general, negative affect was associated with a slowing of the passage of time and positive affect was associated with a relative speeding up of the passage of time. Sadness [3, 4, 8], depression [2, 8], boredom [1, 3, 4] and social isolation [1, 2, 8] were therefore associated with a sensation of time passing more slowly during the pandemic than before, whereas social satisfaction [1, 2], positive mood [1–6] and busyness [1–4] were associated with time passing more quickly than normal. Day to day experience of time during the pandemic was therefore heavily affected by the emotional and social consequences of the measures put in place to reduce transmission.

Whilst it is clear that our day-to-day experience of the passage of time has changed as a result of the pandemic, it remains unclear how we will remember the length of the pandemic in years to come. For example, will the pandemic be remembered as long and slow, which would be consistent with the slowing of the days experienced by many, or short and fast? Understanding memory for the length of the pandemic is important because it’s length is remembered may be an important factor in how it shapes individual and societal identity and recovery [11–14]. For example, trauma is associated with a slowing the passage of time which results in a subjective elongating of the duration of the period of trauma. This lengthening is thought to reinforce trauma and impair recovery [11–14]. Similarly, the subjective lengthening of memories for the length of the pandemic may also make the pandemic seem more recent than in actually was, which may result in feeling of temporal vertigo [11] in which an individual feels lost in timeline of past-present and future. It is therefore important to establish how the length of the pandemic is remembered and how this varies between individuals and over time.

Memory for long durations (e.g. years) is poorly understood due to a lack of research in this area. Studies do however suggest that how long we retrospectively remember an event as lasting for is influenced by memory storage during the period of interest [15]. Epochs from which we can recall many memories are perceived as “long” and epochs from which we can recall few memories are perceived as “short” [15]. Similar effects are observed for contextual change; periods with frequent changes in the environment (high contextual change) are remembered as long whereas periods with few changes in the environment (low contextual change) are remembered as short [16]. The effects are reflected in models of retrospective duration judgements. Ornstein (1969) [15], for example, proposed a “storage-size” model of memory for duration in which the remember duration of an event is a function of the amount of memory stored during that period. Block & Reed (1978) [16] however proposed that remembered duration is specifically a function of contextual change within memory, with periods in which we can recall greater levels of emotional and environmental change being associated with longer perceived duration. This latter theory is supported by evidence that greater executive demands (i.e. working memory, inhibition and attention) are associated with longer remembered durations [17–19].

There is also evidence that retrospective estimates of duration can also be influenced by emotional experience. Thones and Wittmann (2016) [20], for example, demonstrated that retrospective estimates of duration were shorter following mindfulness meditation than music listening. This shortening was explained by increases in relaxation following mindfulness. Similarly, Phillips & Cross (2011) [21] reported that retrospective estimates of the duration of music were influenced by the level of enjoyment evoked by the music, with greater enjoyment being associated with longer retrospective estimates. Johnson & MacKay (2019) [22] also observed a lengthening of the remembered duration of taboo words in relation to neutral worlds. Consistent with Block & Reed [16], one explanation for these results is that greater levels of emotionality, regardless of valence, result in longer retrospective estimates because they result in greater levels of emotional contextual change.

The covid-19 pandemic has resulted in significant changes in day-to-day life, which have often resulted in significant changes in emotion. During periods of social restriction there have been significant changes in working environment/practice and schooling arrangements. There have also been reductions in opportunities for recreation, socialisation and vacation [23] and increases in economic uncertainty [23–25]. Even during periods without national restrictions, daily life has been altered by the long-term societal changes resulting from the pandemic itself, for example, rises in home/hybrid working and disruption to schooling [26–29]. Given these significant changes in daily life, it is plausible memories for the length of the pandemic may be significantly distorted by the events that occurred during the pandemic.

Changes in daily life have also been accompanied by significant changes in mental health and wellbeing during and after the pandemic. Analysis from cross-sectional, cohort and longitudinal studies indicates that for many, mental wellbeing deteriorated during the pandemic [30–35]. Specifically, during periods of lockdown there were increases in feeling of boredom, isolation, anxiety and depression for many [30–35]. There were also significant increases in worry, and to a lesser extent rumination, during periods of lockdown throughout the first year [36]. Indeed a combined analysis of 11 longitudinal studies assessing mental health before and during the first 12 months of the pandemic indicated that psychological distress increased during the initial periods of lockdown, particularly in young adults, and that this increased level of distress was maintained throughout the first 12 months of the pandemic, even during periods of reduced restrictions [37]. This type of prolonged and significant deterioration in mental wellbeing, accompanied by increased worry may also contribute to greater memory load and contextual change during the pandemic, resulting in distortion to memories for the perceived length of the pandemic.

At first glance, reduced opportunities for socialisation, vacation and recreation may mean that fewer memories were formed during the first year of the pandemic than normal. This, according to Ornstein’s [15] memory storage size theory, should result in the pandemic being remembered as subjectively shorter than its actual duration. Similarly, the absence of the commute, conversations with colleagues, the school run and other “out of home” experiences may mean that there was less contextual change during the pandemic than normal. According to Block and Reed’s [16] contextual change model this too would be associated with the pandemic being remembered as relatively short.

Although it is easy to assume that the restrictions during the pandemic would have resulted in fewer memories as less contextual change, it is also plausible that both of these factors actually increased during the pandemic. This is because, the societal changes resulting from the pandemic have necessitated the learning of a significant number of new social rules, such as remembering to wear a mask and stay physically apart, and technical skills, such as online working tools and home schooling. Furthermore, there has also been significant emotional change as a result of the pandemic with incidences of depression, anxiety, eating disorder and stress increasing during the pandemic relative to before [30–37]. Therefore, according to Ornstein’s [15] storage size model and Block and Reed [16] contextual change model, it is plausible that the pandemic may be remembered as longer than its actual duration because we made many new (albeit often unwanted) memories and experienced significant contextual and emotional change.

Initial studies conducted during the first year of the pandemic distortion to the perceived length of the pandemic occurred for many, often resulting in the pandemic being remembered as longer than its actual duration [2]. In the UK for example, 8 months into the pandemic at the start of England’s second lockdown, 54% of participants reported that it felt like longer than 8 months since the start of the first lockdown. Similarly, 62% of participants in Iraq reported that it felt like longer than 11 months since the start of Iraq’s first lockdown [9]. Whilst these studies support suggestions that the pandemic’s duration may be distorted, the perceived length of the pandemic itself was not the focus of either paper and the findings therefore lack sufficient theoretical discussion. Furthermore, neither finding has been replicated in either population and as a result, it is unclear whether the lengthening effects observed would persist later into the pandemic and beyond.

The current study therefore aimed to establish how the length of the first 12 months of the pandemic in the UK was remembered. Specifically, the study aimed to determine whether beliefs about the subjective length were accurate, or whether there were systematic distortions to the remembered length of the first 12 months of the pandemic. Furthermore, the study sought to establish the factors associated with the pandemic being remembered as shorter or longer than its actual duration. These included demographic factors, covid specific factors e.g. shielding status, emotional factors e.g. depression, anxiety and stress, and an estimate of average task-load during the pandemic.

A modified version of the Passage of Time questionnaire developed in Ogden (2020) was used to explore the subjective length of the pandemic 12 months from the imposition of the first UK national lockdown. The questionnaire asked participants to rate the extent to which it felt shorter or longer than 12 months since the first lockdown using a 7-point Likert scale. Participants also completed questions about their anxiety, stress, depression, social satisfaction, physical activity, busyness and compliance with restrictions. Participants also provided demographic details including age, gender, employment status, number of cohabitants and perceived risk from Covid-19.

It was expected that there would be significant distortion to the subjective length of the pandemic because of the significant changes in daily life since its onset. Furthermore, it was expected that the relative distortion to the subjective length of the pandemic would be predicted by the extent to which life had changed due to the pandemic, level of busyness and affective factors. Based on the predictions of Ornstein’s [15] storage size model and Block’s and Reed’s [16] contextual model of retrospective time estimates, it was expected that greater change in life due to the pandemic and greater levels of busyness would be associated with a subjectively longer pandemic because both would be associated with greater memory creation and greater contextual change. It was also expected that greater levels of depression, anxiety and stress would be associated with a subjectively longer pandemic because, as suggested by Block & Reed [16], greater emotionality is associated with greater contextual change. Finally, it was expected that those who perceived themselves to be at greater risk from covid-19, and those advised to shield, would also experience a subjectively longer pandemic due to increased health related stress and anxiety.

## Method

### Participants

1098 participants were recruited through volunteer sampling via email and social media advertising. 332 were excluded from the study because they failed to answer one or more questions, or because they were not currently residing in the UK. This left a final sample of 766 participants (mean age 28.11 years, SD = 12.51) with complete datasets. Table 1 shows demographic information. The study was approved by Liverpool John Moores University Research Ethics Committee (ref 20/NSP/01) and all participants gave informed written consent. The study was conducted in accordance with the principles expressed in the Declaration of Helsinki.

**Table 1:** Subjective ratings of the length of the 12 months since lockdown as a function of demographic factors.

Variable	Group	%	Mean	SD
*Age*	*Young (< 26)*	67.80	4.60	2.03
	*Middle aged*	29.20	4.52	1.86
	*Elder (>60)*	3.00	4.78	1.57
*Gender*	*Male*	33.50	4.58	1.91
	*Female*	65.80	4.58	1.99
	*Other*	0.70	4.40	2.61
*Perceived risk from covid*	*Low*	76.70	4.49	1.97
	*High*	10.10	4.77	2.02
	*Unsure*	13.20	4.92	1.86
*Advised to shield*	*Yes*	10.20	4.46	2.02
	*No*	89.80	4.60	1.96
*Employment*	*Full-time*	19.50	4.46	1.89
	*Part-time*	9.40	4.35	1.81
	*Furloughed*	7.00	4.41	2.07
	*Unemployed*	4.00	4.77	2.09
	*Retired*	2.30	5.22	1.11
	*Student*	55.40	4.65	2.03
	*Disabled*	2.30	4.44	1.82

### Measures

Participants were asked to complete an online survey via Qualtrics (www.qualtrics.com). The survey was made available for 3 weeks (March 15^th^ and April 4^th^ 2021) around the anniversary of the beginning of the first national lockdown (23^rd^ March 2020). At this point England was beginning to exit social and physical restrictions. Households were permitted to meet another household outdoors and schools were open (29^th^ March 2021). This constituted step one in a four-step plan to remove all restrictions by June 2021. The survey recorded demographic information, Covid related information, passage of time judgments, satisfaction with social interactions and level of physical activity. Depression, anxiety and stress were also assessed using the DASS-21 [38], and average daily task load was assessed using a modified version of the NASA-TLX [39]. Participants took approximately 5–10 minutes to complete the questionnaire.

*Perceived length of the pandemic*: participants answered the following question “*It is now 1 year since the beginning of the first national lockdown (23rd March 2020)*. *How long ago does it feel for you*? Responses were provided using a 7-point Likert scale; 1 A lot shorter, 2 somewhat shorter, 3 a little shorter, 4 as normal, 5 a little longer, 6 somewhat longer, 7 A lot longer.

*Demographic questions*: participants were asked to indicate their age, gender, country of residence, employment status, and the number of people in their household (excluding participant themselves).

*Covid related questions*: participants were asked whether they felt at high risk of being hospitalised or dying if they were infected and whether they were shielding.

*DASS-21*: The DASS-21 is a short version of the 42-item Depression Anxiety Stress Scales [38], which measures depression, anxiety, and stress. The questionnaire consists of 3 subscales (depression, anxiety and stress), each containing 7 items (21 items in total). Participants used a 4-point Likert scale to indicate how much each statement applied to them (0: Did not apply to me at all; 1: Applied to me to some degree; 2: Applied to me to a considerable degree; 3: Applied to me very much). A score for each subscale was calculated by summing the score of the items of the respective subscale and multiplied by 2. Each subscale had therefore a score range from 0 to 42. Although the DASS-21 is not a diagnostic tool, however, scores from the DASS-21 can be doubled to enable classification as normal, moderate or severe using the following cut-offs: depression; normal 0–9, moderate 10–20 and severe 21–42, anxiety; normal 0–7, moderate 8–14 and severe 15–42 and stress; normal 0–14, moderate 15–25 and severe 26–42. Cronbach’s alpha for the 21 item DASS questionnaire was 0.95.

*NASA-TLX*: The National Aeronautics and Space Administration-Task Load Index [39] assesses subjective workload using 6 items measuring: mental demands, physical demands, temporal demands, personal performance, effort and frustration. In the current study, a modified version of the NASA-TLX was used to assess the subjective workload of an average day during the last year. Participants were asked to rate each of the six items using a 1–5 Likert scale in which a high score indicated greater task demands. Responses were then summed to obtain a task load score, which ranged from 6 to 30. Cronbach’s alpha for NASA-TLX questionnaire was 0.59

To measure social satisfaction participants were asked to rate “*Since the beginning of the first national lockdown (23rd March 2020)*, *how satisfied are you with your daily level of social interaction*?” using a 5-point Likert scale in which a high score indicated greater satisfaction. To measure physical activity, participants rated “*Since the beginning of the first national lockdown (23rd March 2020)*, *how would you describe your level of physical activity*?*”* Using a 5-point Likert scale in which a high score indicated greater activity. Finally, participants also used a 5-point Likert scale to rate to what extent they agreed that: "*My current daily routine is very different to how it was before Covid-19*”. Here, a high score indicated greater agreement.

### Data analysis

The main dependant variable, perceived length of the year, was an ordinal scale. Consequently, non-parametric analyses were conducted. Kruskal-Wallis and Mann-Whitney U tests were used to establish the effect of age (divided in young, middle aged and elder as in Ogden, 2020 [1]), gender, personal risk and shielding status. To assess the relationship between the passage of time and measures of affect (DASS-21 depression, anxiety and stress scores, satisfaction with social interaction), task load (NASA-TXL scores and rating of physical activity), compliance with lockdown, change of life over the year and age, Spearman’s correlations were conducted. Finally, to assess whether these factors were predictive of the perceived length of the year, ordinal logistical regression analysis was conducted. Throughout, Bonferroni correction for multiple comparisons was applied.

## Results

**Fig 1:**
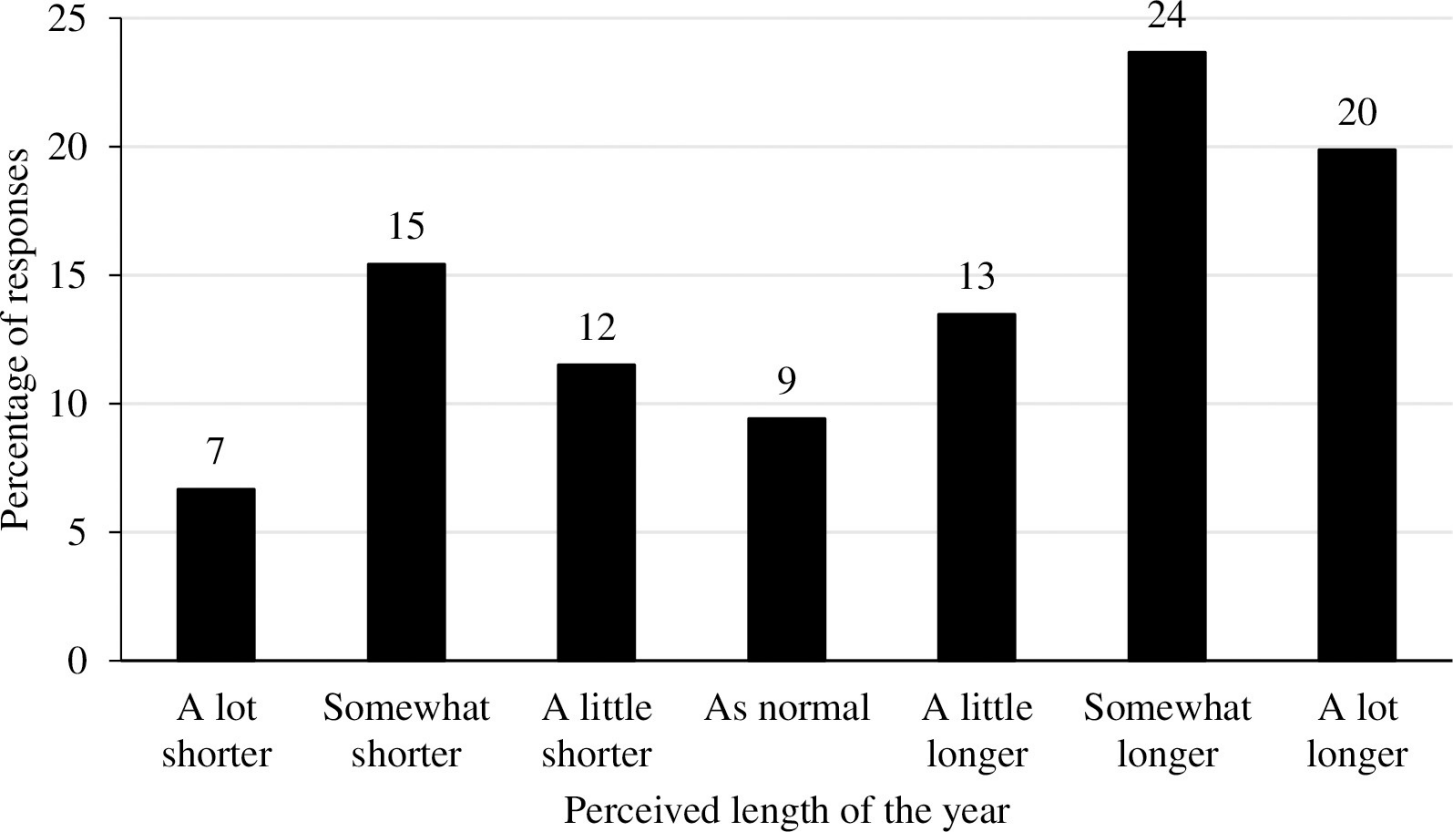
The frequency of responses for each Likert point for the perceived length of the year since lockdown began.

Examination of Fig 1 suggests that there was significant distortion to the subjective length of time which had passed since the first UK lockdown began. Only 9% of participants felt that the 12 months felt like 12 months. 34% reported that it felt like a shorter period than 12 months. 57% reported that it felt subjectively longer than 12 months. Subjective lengthening of the perceived length of the 12 months since lockdown was therefore the predominant direction of distortion.

Table 1 shows the mean subjective length of the 12 months since lockdown began as a function of demographic factors. Examination of Table 1 suggests the subjective length of the pandemic was similar cross genders, age groups, employment types, shielders and non-shielders and perceived vulnerability to covid-19.

Kruksall Wallis tests confirmed no significant effect of age group *H*(2) = .67, *p* = .72, gender *H*(2) = .005, *p* = .98, perceived personal risk *H*(2) = 5.16, *p* = .08 or employment status *H*(6) = 5.59, *p* = .47 on the subjective length of the first year since lockdown. A Mann Whitney U test confirmed no significant effect of shielding status on the subjective length of the first year since lockdown *U* = 27296.00, *p* = .59.

The relationship between the subjective length of the year, depression, anxiety, stress, change to daily life as a result of covid-19, number of cohabitants, compliance with restrictions and task load was assessed using Spearman’s correlations (Table 2). Examination of Table 2 suggests that a subjective lengthening of the preceding 12 months was associated with greater levels of depression, anxiety and stress. The subjective length of the preceding 12 months was not related to the number of cohabitants, compliance with regulations or task load. Greater depression was associated with greater anxiety and stress. Greater anxiety was also associated with greater stress. Compliance with regulations was negatively associated with the number of cohabitants and positively related to task-load.

**Table 2:** Spearman’s correlation coefficients for the relationship between the perceived length of the year, affect, number of cohabitants, task load and compliance, social satisfaction and physical activity.

	*Depression*	*Anxiety*	*Stress*	*Life change*	*N of cohabitants*	*Compliance*	*Task load*	*Social Satisfaction*	*Physical activity*
*Subjective length*	.22**	.17**	.20**	.13**	.03	-.04	-.008	.22**	.03
*Depression*		.70**	.78**	.14**	.10	-.06	-.09	.43**	-.28**
*Anxiety*			.74**	.09	.08	.02	-.001	.27**	-.28**
*Stress*				.18**	.08	.002	.05	.39**	-.25**
*Life change*					-.05	.01	-.02	.27**	.11
*N of Cohabitants*						-.16**	-.09	-.01	.06
*Compliance*							.16**	-.07	.05
*Social Satisfaction*									-.21**

** *p* < .001

Ordinal logistic regression with proportional odds was conducted to establish the effect of demographic factors, measures of affect and task load, social satisfaction, physical activity, change of life and compliance on the subjective length of the preceding 12 months. Table 3 shows the odds ratios for each variable with 95% confidence intervals. The model was a statistically significant, χ^2^(21) = 85.64, *p* < .001 fit for the data, with pseudo R squared values of .03 - .11. Table 3 shows that the subjective length of the preceding 12 months was predicted by satisfaction with social interaction, physical activity, change of routine, depression, anxiety and shielding status. Feeling like the last 12 months was longer than 12 months was therefore associated with greater depression and anxiety, lower levels of satisfaction with social interaction, lower levels of physical activity, greater change in daily routine and shielding.

**Table 3:** Ordinal logistic regression with odds ratio (and 95% of confidence intervals) with subjective length of the preceding 12 months as outcome and age, depression, anxiety, stress, compliance, social satisfaction, physical activity, number of cohabitants, task load, daily routine, gender, shielding status, covid perceived risk and employment status as predictors.

		*Wald*	*Odds Ratio*	*95% CI*
Age		1.31	1.01	.99–1.02
Depression		4.28	1.04*	1.00–1.08
Anxiety		4.04	1.05*	1.00–1.10
Stress		.001	1.00	.96–1.05
Compliance		1.48	.09	.77–1.07
Social Satisfaction		16.10	1.33**	1.16–1.53
Physical activity		5.20	.87*	.78-.98
N. Cohabitants		.81	1.04	.14–3.19
Task Load		.04	1.00	.96–1.04
Daily Routine		3.90	1.16*	1.01–1.34
Gender	Male	.27	1.51	.31–7.31
	Female	.14	1.35	.28–6.45
	Other (reference)			
Shielding status	Yes	4.39	.61*	.38 - .97
	No (reference)			
Perceived personal risk	Yes	.06	1.08	.61–1.91
	No	3.17	.70	.47–1.04
	Unsure (reference)			
Employment	Full-time	.68	1.46	.60–3.55
	Part-time	.14	1.19	.47–3.02
	Furloughed	.01	.96	.37–2.51
	Unemployed	.90	1.66	.58–4.76
	Retired	.50	1.56	.46–5.33
	Student	.46	1.35	.57–3.16
	Disabled (reference)			

** *p* < .001

* *p* < .05.

## Discussion

The current study sought to establish how people remembered the subjective length of the first 12 months since lockdown was initiated in the UK. It also sought to establish the factors which were associated with the pandemic being remembered as “shorter” or “longer” than 12 months. The results show that very few people agreed that it felt like 12 months since the first lockdown began. The overwhelming majority of people therefore experienced some temporal distortion to their memory representation of how long the year had lasted. Although some people (34%) felt like it had been less than 12 months since the start of lockdown, the majority (57%) felt like it was longer. For most people, the first year of the pandemic therefore felt subjectively longer than its actual duration. This replicates earlier findings from England’s second lockdown [2] and 11 months into Iraq’s pandemic [9]. The tendency for people to report experiencing the first 12 months of the pandemic as longer or shorter than its actual duration is perhaps unsurprising given the tendency for people to experience distortions to time during the days and weeks of the pandemic [1–10]. The changes in life as result of covid have therefore not only affected our short-term subjective experience of duration, but also our long-term representations of the pandemic’s length.

How long the first 12 months was felt to last was predicted by depression, anxiety, satisfaction with social interactions, the extent to which life had changed as a result of the pandemic, levels of physical activity and shielding status. Feeling like it was longer than 12 months since the first lockdown was therefore associated with greater depression and anxiety, reduced satisfaction with social interaction, greater change of life, reduced physical activity and being advised to shield. Feeling like it was less than 12 months was therefore associated with lower levels of depression and anxiety, greater social satisfaction and physical activity, less life change as a result of covid and not being advised to shield. Age, stress, compliance with restrictions, task load, number of cohabitants, perceived personal risk, gender and employment were not significant predictors of the subjective length of the first year since lockdown.

These findings do not offer universal support for memory storage size and contextual change-based models of retrospective duration [15–18]. This is because numerous factors which could be considered to be indexes of memory formation and contextual change were not predictive of the perceived length of the first 12 months since lockdown. Both the contextual change and memory formation models would predict that greater task loads should result in longer perceptions of time because more memories would be formed and more contextual change would take place when busy then when not busy. Task load was not however associated with the perceived length of the 12 months. Similarly, shielding, which we speculate would be associated with few memory formations and less contextual change due to the requirement to stay in at home and avoid contact with others, was associated with a lengthening of the 12 months rather than a shortening. Furthermore, greater dissatisfaction with levels of social interaction, which for many means insufficient social interaction and thus fewer social memories, was associated with a lengthening rather than a shortening as would be predicted. The factors which influence how long we remember a period to last for are therefore more complex than simply how many memories we have made and how much contextual change we experience.

The finding of the current study instead suggest that affective factors are critical to our memory of the subjective length of long events. In particular, negative affect, i.e. depression anxiety and social dissatisfaction, appears to be associated with a lengthening of perceived duration whereas as positive affect is associated with a shortening of perceived duration. The effects of affective valence on the remembered length of an event are comparable to those observed for passage of time judgements and prospective time judgements conducted in laboratory settings. When judging the subjective speed at which time is passing for example, negative affect is associated with a slowing of time and positive affect a speeding up of time [1–10, 40, 41]. Prospectively, positive affect is associated with a shortening of time whereas negative affect is associated with a lengthening of perceived time [42–44]. Emotional experience therefore appears to be a critical factor in the experience of distortions to a range of temporal experiences and judgments.

One critical issue is how our memory for the subjective duration of the pandemic influences individual and societal recovery from the pandemic. During periods of trauma, a slowing of the passage of time often occurs which results in a subjective lengthening of the period of trauma [1–9, 11–14]. This can also result in the period of trauma being recalled as longer and more recent than it actually was [1–9, 11–14]. The term temporal vertigo is used to describe the experience of confusion and anxiety about where and when we belong in the timeline of past, present and future [11]. Temporal vertigo occurs during and after crises and can influence the extent to which individuals and societies conceptualise and recover from a crisis. How long we remember the pandemic as lasting for may therefore influence the extent to which we experience temporal vertigo in relation to the pandemic. We speculate that people who remember the pandemic as being longer than its actual duration may be more at risk of temporal vertigo. This is because a longer pandemic may feel more recent and thus more present. Similarly, those who remember the pandemic as short may be at less risk of temporal vertigo because, by being over subjectively faster, the pandemic perhaps feels less present and more past.

At present, our understanding of how memory for the length of pandemic may influence recovery from the pandemic is limited. Whilst it is clear that temporal experience during and after the pandemic are related to emotional and social states during and after the pandemic, the specific mechanisms by which temporal experience facilitate or impair recovery are unknown. However, emergent evidence of a time-trauma cycle in which distortions to time act as a reinforcer to trauma coupled with evidence that the deleterious effects of the pandemic on mental-wellbeing are long lasting [36, 37] highlights the need for further research into the role of temporality in trauma. Researchers should therefore seek to understand how individual temporal experience and memory for time contribute towards impaired recovery and reduced wellbeing, and how these effects can be mitigated by methods known to reduce the perceived duration of events [45, 46]. Similarly, mental health practitioners should be mindful that inaccurate memory for the duration of a traumatic event is common and indeed appears to be an inherent feature of trauma itself. Future developments of trauma therapy should therefore explore the utility of interventions aimed at reducing the remembered duration of trauma as a mechanism to improve wellbeing.

### Limitations

The NASA-TLX was designed and validated as an index of task-load following a singular discrete task [39]. In the current study however, a modified version of the NASA-TLX was employed in which participants estimated their average task-load on a typical day during the first 12 months of the pandemic. This modification was based on previous research in which the NASA-TLX was used as an estimate of workload over longer periods of time, for example, by nurses [47], car production workers [48], diabetes patients [49] and control room operators [50] to estimate workload across a whole work shift. Although the use of the NASA-TLX as an estimate of task-load for whole day activities has been validated [49], it is possible that the retrospective average estimate used in the current study did not provide accurate or reliable measures of actual task-load during the pandemic.

Existing theories suggest that the storage size of memories formed during an epoch are determinant of the perceived length of the epoch [15–19]. These conclusions are based on laboratory studies which have directly manipulated the number of events encoded into memory during a task and then compared estimates of duration for the task [17]. Although the current study tentatively concludes memory storage is not a determinant factor in that real-world retrospective duration judgments it is important to note that the current study did not take a direct measure of number or size of memories formed during the pandemic. It is not therefore possible for the current to study to exclude memory storage size as a predictor of remembered duration. Future studies may therefore wish to consider analysis of diary entries during the pandemic to provide a more direct measure of memory formation during that time.

The current study also failed to measure participants retrospective and episodic memory function. If retrospective estimates of long epochs are based on the number of memories stored during the epoch, memory function may have significant influence on perceived duration. In particular, impaired memory function may distort retrospective estimates of duration, making events seem shorter than they were because fewer memories can be recalled. Future research should therefore explore the influence of memory function on retrospective duration estimates.

Finally, the use of an opportunity sampling method resulted in a sample which was not representative of the population. In particular, the small number of people aged 65 years and above and the relatively smaller proportion of male to female participants may have limited the conclusions which can be drawn about the experiences of people in these groups.

### Conclusions

For most people, the subjective length of the first year since lockdown began is highly distorted. The majority of people remember the first year as feeling like it was longer than 12 months suggesting a lengthening of the subjective duration of the pandemic. Feeling like the pandemic was longer than its actual duration was associated with negative affect (depression, anxiety and social dissatisfaction), greater change of life, reduced physical activity, and shielding. Feeling like the pandemic was shorter than its actual duration was associated with more positive affect, greater activity, reduced life change and not shielding. Emotional experience therefore appears to be a primary determinant of the pandemics perceived duration. Future research should aim to establish how the subjective of the pandemic, and the extent to which it is in the “past” influences wellbeing, coping and recovery.

## Supporting information

S1 Data (SAV)
